# The retrosigmoid trans–middle cerebellar peduncle approach for pontine cavernous malformations: multicenter experience

**DOI:** 10.1007/s00701-026-07043-7

**Published:** 2026-09-22

**Authors:** Shadi Al-Afif, Elvis J. Hermann, Anton Früh, Julius Oltmanns, Johannes Woitzik, Peter Vajkoczy, Joachim K. Krauss

**Affiliations:** 1 Department of Neurosurgery, Hannover Medical School, Hannover, Germany; 2 Department of Neurosurgery, Charité Universitätsmedizin Berlin, Berlin, Germany; 3 Department of Neurosurgery, Carl Von Ossietzky University Oldenburg, Oldenburg, Germany

**Keywords:** Middle cerebellar peduncle, Brainstem cavernoma, Cavernous malformation, Trans–middle cerebellar peduncle approach, Brainstem, Pons

## Abstract

**Background:**

Intrinsic pontine cavernous malformations represent one of the most challenging entities in neurosurgery because of their deep location and close proximity to critical brainstem nuclei and long tracts. Traditional surgical approaches are associated with considerable morbidity due to transgression of eloquent neural tissue. The identification of the trans-middle cerebellar peduncle (trans-MCP) approach, has enabled safer access to selected pontine lesions. However, apart from publications of the center where the trans-MCP approach was introduced (Phoenix), little is known about its use and surgical outcome.

**Methods:**

Patients with symptomatic intrinsic pontine cavernous malformations were treated via a retrosigmoid trans-MCP approach at three academic neurosurgical centers. Data were obtained from institutional databases and included clinical presentation, imaging characteristics, surgical details, and follow-up outcomes.

**Results:**

Five patients (3 men, 2 women; mean age 44.8 years) were included. All lesions were intrinsically located within the pons without pial or ependymal surface presentation and demonstrated typical hemorrhage MRI characteristics with mass effect on adjacent structures. The median preoperative mRS was 4 (range 1–4), and recurrent hemorrhage before surgery occurred in 3/5 patients. The retrosigmoid trans–MCP approach was performed with neurophysiological monitoring. Gross total resection was achieved in all instances. Postoperative neurological morbidity occurred in all five patients but was predominantly transient. At follow-up (range 4–14 months), no residual or recurrent cavernous malformations were detected on MRI. Neurological status had improved in all cases. Residual symptoms were minor and included mild ataxia, facial hypesthesia, dysarthria, or vertigo. Functional outcomes were favorable, with a median follow-up mRS of 1 (range 1–2).

**Conclusion:**

The retrosigmoid trans–MCP approach represents a relatively safe, effective, and reproducible surgical strategy for selected pontine cavernous malformations. When performed by experienced surgeons, this approach can be successfully implemented across specialized centers.

## Introduction

Pontine cavernous malformations account for approximately 20–35% of all brainstem cavernomas and usually present with hemorrhage, cranial nerve deficits, ataxia, dysarthria, and long tract symptoms [31]. Historically, neurosurgeons avoided operative treatment unless absolutely necessary because early attempts at resection were associated with high rates of permanent disability [13, 20, 30]. Indications for surgery include hemorrhage and neurological deterioration resulting from lesion-related mass effect [3, 4, 14, 22, 25, 34]. The presence of a pial or ependymal surface component is a crucial factor in determining the optimal operative corridor, as it reduces procedure-related morbidity by limiting the need to traverse eloquent neural tissue.

Brainstem cavernous malformations that do not reach a natural surface (i.e., intrinsically located lesions) have traditionally been regarded as inoperable [18]. Nevertheless, their natural history—especially the high risk of recurrent hemorrhage and progressive neurological decline—supports a more aggressive surgical strategy in carefully selected symptomatic patients [21, 31]. These challenges motivated the identification of “safe entry zones” (SEZs), a concept formalized to reduce the risk of transgressing eloquent tissue [10]. A major breakthrough occurred when Hebb & Spetzler described the lateral transpeduncular safe entry zone, utilizing the trans middle cerebellar peduncle (trans-MCP) as a more tolerant entry point [18].

The trans-MCP approach provides a feasible surgical corridor for pontine cavernous malformations [16]. Its utility stems from the fact that it avoids direct transgression of densely packed brainstem nuclei and critical longitudinal fiber pathways—including the corticospinal tract, the medial longitudinal fasciculus, and the medial lemniscal system—thereby substantially reducing the risk of postoperative neurological morbidity. However, apart from the series published by the groups of Spetzler and Lawton [1, 2, 5–9, 16, 19] and few case reports [15, 17, 27, 28, 33], there remains limited knowledge about the use of the trans-MCP approach for intrinsic pontine cavernous malformations. We therefore decided to present the experience with this approach in three large academic centers.

## Methods

### Study design and patient cohort

Subsequent to a video presentation of the trans-MCP approach (JKK) upon the occasion of the German Society of Neurosurgery annual congress in 2025, participants were asked whether or not they have experience with this approach. Only two other centers indicated that they had used this strategy recently. Inclusion criteria for the present series were (1) symptomatic hemorrhagic pontine cavernous malformation, (2) intrinsic lesion location without pial or ependymal surface presentation, and (3) trans-MCP approach without variations. Clinical, radiological, surgical, and follow-up data were collected and analyzed, including presenting symptoms, lesion characteristics, operative details, extent of resection, and functional outcome assessed by the modified Rankin Scale (mRS; 0 = no symptoms, 1–2 = no significant disability, 3–5 = moderate to severe disability, 6 = death).

### Radiological assessment

Preoperative magnetic resonance imaging (MRI) and computed tomography (CT) scans were reviewed to assess lesion location, hemorrhage characteristics, size, and mass effect on adjacent brainstem structures. Postoperative imaging included early CT within 6 h after surgery and follow-up MRI to evaluate extent of resection and detect residual or recurrent cavernous malformations.

### Surgical technique

All procedures were performed under continuous neurophysiological monitoring, including motor evoked potentials (MEP), somatosensory evoked potentials (SEP), and electromyographic surveillance of cranial nerves to ensure real-time assessment of brainstem function. Neuronavigation was used for trajectory planning and intraoperative orientation. While the retrosigmoid craniotomy provides the access, the most important step of the approach is dissection of the horizontal (petrosal) fissure with exposure of the MCP, which widens the corridor and permits a posterolateral peduncular approach [23, 27]. In addition to defining the entry point, neuronavigation is of considerable practical value in maintaining the correct orientation through the MCP toward the cavernoma.

Patients were positioned in either a prone or supine position depending on the center. The head was secured in a Mayfield clamp and turned to the ipsilateral side in the prone position or to the contralateral side in the supine position, and slightly flexed to optimize the operative angle toward the cerebellopontine angle and the MCP. After sterile preparation and draping, a skin incision was made posterior to the auricle. A retrosigmoid, suboccipital craniotomy was performed to expose the margins of the transverse and sigmoid sinuses. Following dural opening, cerebrospinal fluid was released to facilitate cerebellar relaxation and to prevent fixed retraction. A brain spatula was used to gently retract the cerebellar hemisphere. The arachnoid membrane over the cerebellopontine angle was sharply opened, allowing visualization of key anatomical landmarks including the trigeminal nerve root, the facial–vestibulocochlear complex (VII/VIII), and the flocculus. The horizontal (petrosal) fissure between cranial nerve V and the VII/VIII complex was then sharply split, allowing the superior and inferior petrosal surfaces to fall away and expose the posterolateral MCP surface behind the trigeminal root-entry zone [19, 27]. After confirming the planned entry point with neuronavigation, —selected laterally on the MCP and aligned with the long axis of the lesion—a small opening was made within the MCP safe-entry zone. Microneurosurgical dissection then proceeded along the natural transverse fibre orientation of the peduncle, remaining within the ventral two-thirds of the pons to spare the corticospinal tract anteriorly and the pontine tegmentum posteriorly, thereby fashioning a structured corridor toward the lesion without undue manipulation of surrounding pontine tissue [16]. Once the cavernous malformation was reached, hematoma contents were evacuated, and the cavernoma capsule was progressively mobilized and removed in a piecemeal fashion. Resection was carried out under continuous neuromonitoring surveillance to avoid injury to adjacent eloquent structures. Care was taken to preserve developmental venous anomalies when present. After removal of the cavernoma, the resection cavity and MCP corridor were carefully inspected for hemostasis and residual pathology. A watertight dural repair was achieved using sutures and reinforced with the placement of a collagen sealant patch coated with human fibrinogen and human thrombin (TachoSil®). Cranial reconstruction was completed with polymethylmethacrylate (PALACOS®) and miniplate fixation. The fascia and skin were closed in a multilayer fashion. Postoperatively, patients were transferred to the intensive care unit for overnight monitoring.

### Postoperative management and follow-up

Early postoperative CT imaging was routinely performed within 6 h. Neurological status was assessed serially, with particular focus on cranial nerve function, motor deficits, and cerebellar signs. Patients were subsequently transferred to the regular ward and, after a few days, to specialized neurorehabilitation facilities for further treatment and functional recovery. Follow-up was performed on an outpatient basis 3–6 months after discharge from neurorehabilitation and included MRI and comprehensive neurological examination.

## Results

### Cohort characteristics

Five patients (3 men, 2 women; mean age 44.8 years, range 27–61 years) with symptomatic, hemorrhagic pontine cavernous malformations were included in this study and treated surgically via the retrosigmoid trans-MCP approach. Two patients had surgery at Hannover Medical School (MHH), two at the Charité Berlin, and one at Carl von Ossietzky University, Oldenburg. All patients presented with clinically significant neurological deficits, encompassing cranial nerve dysfunction, long-tract signs, and cerebellar symptoms. Disease severity at presentation was substantial, with a median preoperative modified Rankin Scale (mRS) score of 4 (range 1–4). Recurrent hemorrhage before surgery occurred in three of five patients (60%), reflecting a progressive clinical course. Time to surgery varied widely, ranging from acute intervention (3 days) to delayed treatment (18 months), indicating heterogeneity in disease evolution and surgical timing (Table 1).

**Table 1:** Demographic, clinical, and radiological characteristics of the study cohort

Case	Age	Sex	Initial clinical presentation	Clinical course	Location	MRI characteristics	Volumen of the hemorrhagic cavernoma (cm^3^)	Preoperative mRS	Time interval between diagnosis and surgery
1	61	M	Vertigo, diplopia, gait ataxia	Left hemiparesis, (4 +/5), left facial paresis, vertical gaze palsy, dysarthria	Central pontine hemorrhage with dorsal extension into the pontine tegmentum	Subacute hemorrhage with heterogeneous signal intensity, surrounding edema, and mass effect with compression of the fourth ventricle and the right corticospinal tract	9.7	4	3 days
2	27	M	Right hemihypesthesia, vertigo, gait ataxia, hearing impairment with tinnitus	Rebleeding 4 days after the first presentation with right abducens paresis, right facial paresis, dysarthria, left hemiparesis (4/5)	Central pontine hemorrhage with extension into the proximal right middle cerebellar peduncle and dorsally toward the pontine tegmentum	Subacute hemorrhage with heterogeneous signal intensity surrounding edema, and mass effect with compression of the fourth ventricle	11.8	4	9 days
3	58	F	Headache, vertigo, mild glossopharyngeal paresis, left hemiparesis(+ 4/5), gait ataxia	Rebleeding 6 days after the first presentation with marked dysarthria and worsening the gait ataxia	Central pontine, extending into the right cerebral peduncle with smaller eccentric components left	Subacute hemorrhage with herterogeneous signal internsitiy, surrounding edema, no narrowing of cerebrospinal fluid pathways	3.8	4	9 days
4	33	F	Headace, diplopia right hemihypesthesia involving the face, distal arm, and distal leg hemiparesis (4/5)	One week of inpatient treatment with near-complete resolution of symptoms; persistent mild paresthesia of the left arm and leg. Follow-up imaging 1,5 years later demonstrated rebleeding without new clinical symptoms, leading to the decision for surgical intervention	Right pontine and dorsally toward the pontine tegmentum	Hemorrhage presenting as a multilobulated lesion with heterogeneous signal intensity, a surrounding hemosiderin rim, cystic components with fluid–fluid levels	2.2	1	18 months
5	45	M	Headache, nausea, vertigo, gait ataxia, right-sided hemihypesthesia involving the face, distal arm, and distal leg	Mild improvement of symptoms, followed by the decision for surgical intervention	Left pontine	Subacute hemorrhage	2.7	3	26 days

### Lesion characteristics

**Fig. 1:**
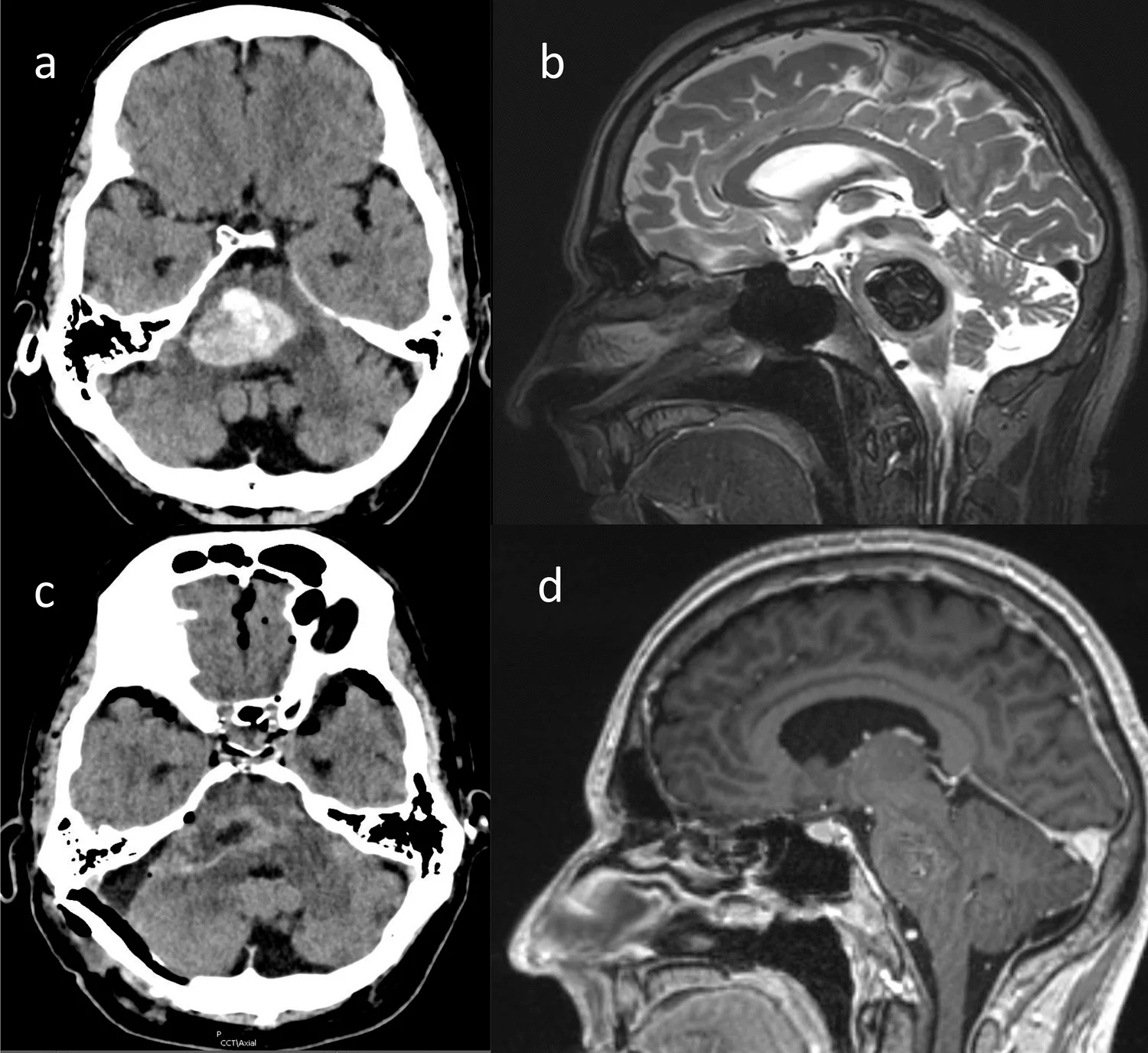
Case 1. **a** Preoperative native cranial CT demonstrating an acute pontine hemorrhage, highly suspicious for an underlying pontine cavernous malformation. **b** Preoperative cranial MRI with T2-weighted sequences showing a large intrapontine hemorrhage with pronounced compression of the pons and surrounding edema. **c** Early postoperative native cranial CT illustrating marked decompression of the pons and delineating the surgical trajectory of the trans-MCP approach. **d** Postoperative MRI demonstrating significant relaxation of the pons with no evidence of residual cavernous malformation

**Fig. 2:**
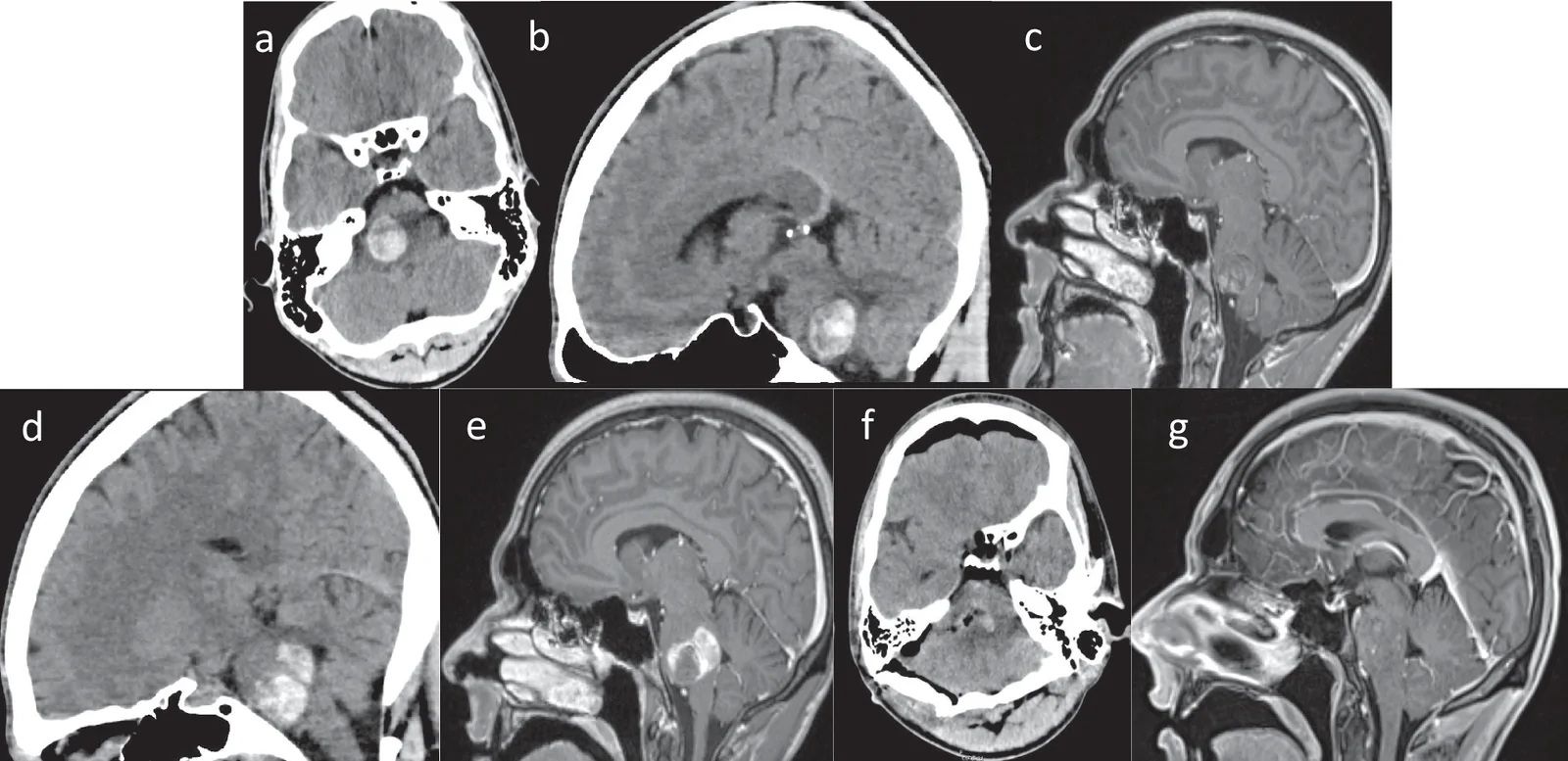
Case 2. **a**, **b** Initial cranial CT demonstrating an acute pontine hemorrhage, more pronounced on the right side. **c** Preoperative MRI, including T1-weighted sequences, showing hemorrhage at different stages, highly suggestive of an underlying pontine cavernous malformation. **d**, **e** Follow-up cranial CT and contrast-enhanced T1-weighted MRI obtained after clinical deterioration, demonstrating recurrent hemorrhage of the cavernoma with cranial and dorsal extension. **f** Early postoperative cranial CT illustrating the postoperative status after hematoma evacuation and delineating the surgical trajectory of the trans-MCP approach. **g** Postoperative MRI demonstrating effective decompression of the brainstem without evidence of residual cavernous malformation

All five cavernous malformations were intrinsically located within the pons, with central positioning and variable extension into the pontine tegmentum and/or the MCP. None of the lesions demonstrated pial or ependymal surface presentation. MRI revealed the hemorrhagic signal characteristics of cavernous malformations, including heterogeneous signal intensity and perifocal edema. A mass effect on critical structures — including the fourth ventricle and the corticospinal tract — was observed in all patients. Hemorrhagic cavernoma volumes ranged from 2.2 to 11.8 cm^3^, with a median size of 3.8 cm^3^, representing moderate to large brainstem cavernomas (Table 1, Figs. 1 and 2).

### Preoperative clinical presentation

The preoperative neurological profiles varied considerably across patients, reflecting the extent of pontine involvement. Case 1 (61-year-old man, Hannover Medical School) presented with vertigo, diplopia, and gait ataxia, subsequently developing left hemiparesis (MRC grade 4 +/5), left facial nerve paresis, vertical gaze palsy, and dysarthria. Case 2 (27-year-old man, Hannover Medical School) initially presented with right-sided hemihypesthesia, vertigo, gait ataxia, and hearing impairment with tinnitus; four days later, neurological deterioration occurred with right abducens paresis, right facial paresis, dysarthria, and left hemiparesis (MRC grade 4/5) secondary to recurrent hemorrhage. Case 3 (58-year-old woman, Carl von Ossietzky University, Oldenburg) presented with headache, vertigo, mild glossopharyngeal paresis, left hemiparesis (MRC grade 4 +/5), and gait ataxia, with recurrent hemorrhage six days later causing dysarthria and worsening gait ataxia. Case 4 (33-year-old woman, Charité Berlin) experienced headache, diplopia, right-sided hemihypesthesia involving the face, distal arm, and distal leg, as well as left hemiparesis (MRC grade 4/5); after near-complete resolution of symptoms with conservative management, repeat imaging 1.5 years later revealed rebleeding prompting surgical decision-making. Case 5 (45-year-old man, Charité Berlin) presented with headache, nausea, vertigo, gait ataxia, and right-sided hemihypesthesia of the face, distal arm, and distal leg, followed by mild spontaneous improvement before surgical intervention was planned (Table 1).

### Surgical performance and feasibility

The retrosigmoid trans-MCP approach was successfully implemented in all five patients without the need for conversion to an alternative surgical corridor. Cases 1 and 2 were operated in the prone position, while Cases 3, 4, and 5 were placed in a supine position. Intraoperative neuronavigation was employed in all cases. Multimodal intraoperative neurophysiological monitoring — comprising motor evoked potentials (MEP), somatosensory evoked potentials (SEP), and cranial nerve electromyography (EMG) — was applied in all patients, with the exception of Case 3, in whom SEP monitoring was not utilized. Gross total resection was confirmed by postoperative MRI in all five patients demonstrating the technical feasibility and reproducibility of the trans-MCP corridor across the different tertiary neurosurgical centers and surgical settings (Table 2).

**Table 2:** Surgical characteristics and postoperative outcomes

Case	Surgical positioning	Intraoperative navigation	Intraoperative monitoring	Extent of resection (post-op MRI)	Postoperative outcome	Duration of hospital stay (days)	Follow-up duration (months)	Last follow-up MRI	Neurological outcome at follow-up	mRS at follow-up
1	Prone	Yes	MEP, SEP, cranial nerve EMG	Complete resection	Improvement of hemiparesis and cranial nerve deficits; right facial hypesthesia. and slight ataxia of the right arm, transient dysphagia	7	10	No residual or recurrence of cavernoma	Mild ataxia of the right hand, right facial hypesthesia	1
2	Prone	Yes	MEP, SEP, cranial nerve EMG	Complete resection	Improvement of hemiparesis, complete resolution of tinnitus with improved hearing, worsening dysarthria	7	14	No residual or recurrence of cavernoma	Mild gait ataxia and dysarthria	2
3	Supine	Yes	MEP, cranial nerve EMG	Complete resection	Dysphagia (requiring parenteral nutrition), right facial hypesthesia, persistent gait ataxia and dysarthria	5	4	No residual or recurrence of cavernoma	Mild gait ataxia, right facial hypesthesia	1
4	Supine	Yes	MEP, SEP, cranial nerve EMG	Complete resection	Hypesthesia involving all three divisions of the right trigeminal nerve with reduced blink reflex, vertigo and nausea	10	12	No residual or recurrence of cavernoma	Right facial hypesthesia	1
5	Supine	Yes	MEP, SEP, cranial nerve EMG	Complete resection	Persistent right-sided nystagmus on right and upward gaze, vertigo	7	6	No residual or recurrence of cavernoma	Vertigo	1

### Postoperative morbidity

New neurological deficits attributable to MCP transgression occurred in all patients; however, these were predominantly transient and did not prevent functional independence at follow-up. Case 1 experienced right facial hypesthesia, slight ataxia of the right arm, and transient dysphagia in the early postoperative period; however, the preoperative hemiparesis and other cranial nerve deficits showed marked and rapid improvement. Case 2 demonstrated transient worsening of dysarthria postoperatively, alongside complete resolution of tinnitus and improvement in the left hemiparesis and hearing. Case 3 developed dysphagia requiring temporary parenteral nutritional support along with right facial hypesthesia, gait ataxia and dysarthria. Case 4 experienced hypesthesia involving all three divisions of the right trigeminal nerve with a reduced blink reflex, as well as vertigo and nausea. Case 5 had right-sided nystagmus on rightward and upward gaze, accompanied by vertigo. Median length of hospital stay was 7 days (range 5–10 days, Table 2).

### Outcome at follow-up

At mid-term follow-up (range 4–14 months), no residual or recurrent cavernous malformation was detected on MRI (see Figs. 1 and 2). A consistent pattern of neurological improvement was observed relative to the immediate postoperative status, particularly with regard to preoperative hemiparesis and cranial nerve deficits. Residual symptoms at follow-up were minor across the cohort. Case 1 exhibited mild ataxia of the right hand and right facial hypesthesia (mRS 1). Case 2 demonstrated mild residual gait ataxia and dysarthria (mRS 2). Case 3 showed mild gait ataxia and right facial hypesthesia (mRS 1). Case 4 demonstrated mild residual hypesthesia of the right side of the face (mRS 1), a remarkable recovery given the initial trigeminal distribution of deficits. Case 5 reported persistent vertigo as the sole residual symptom (mRS 1). Functional outcome was favorable in all patients, with a median follow-up mRS of 1 (range 1–2), corresponding to full independence in activities of daily living. (Table 2).

## Discussion

Intrinsic pontine cavernous malformations remain among the most formidable challenges in neurosurgery. The pons harbors multiple vital structures within a very limited spatial range, including the corticospinal tracts, pontine nuclei, cranial nerve nuclei, and essential ascending sensory pathways, as well as other critical structures such as the medial longitudinal fasciculus and the reticular formation. These anatomical constraints explain the historically high morbidity associated with surgical treatment of pontine lesions [13, 20, 30].

Early surgical approaches—most notably trans–fourth ventricular routes—frequently violated eloquent neural tissue in order to access the lesion, often resulting in new neurological deficits such as facial nerve paresis, abducens nerve paresis, vertical gaze palsy, hemiparesis, dysarthria, or dysphagia. Even with the use of intraoperative neurophysiological monitoring, these approaches carry a substantial risk of injury, as surgical manipulation occurs only millimeters away from highly sensitive neural structures.

One of the most notable contributions was provided by Hebb and Spetzler [18], who described the lateral transpeduncular approach, utilizing the MCP as a more tolerant entry point for deep pontine lesions. A similar approach was also described by Ohue et al., who termed it the retrosigmoid suprafloccular transhorizontal fissure approach, demonstrating that splitting the horizontal (petrosal) fissure allows exposure of the MCP with minimal cerebellar retraction [23]. Kalani et al. further refined this strategy with the retrosigmoid petrosal fissure transpeduncular approach, emphasizing that fissure dissection improves surgical exposure and shortens the operative trajectory to central pontine lesions while minimizing disturbance of the corticospinal tract [19].

The trans-MCP corridor is best suited for intrinsic cavernous malformations of the lateral and central pons that lack a pial or ependymal surface presentation, since under these circumstances it offers the most tolerant safe-entry zone [16, 18]. Planning the trajectory along the long axis of the lesion and keeping the dissection within the ventral two-thirds of the pons—within the white-matter "invisible triangle" bounded by the corticospinal tract anteriorly, the superior cerebellar peduncle superomedially, and the pontine tegmentum posteromedially—protects the eloquent tracts and nuclei [16]. By angulating the corridor, the approach also reaches lesions with limited rostral (midbrain), caudal (medulla), or posterior (rhomboid) extension [16]. Its limits are defined by the same anatomy: lesions extending into the contralateral midbrain require a steep superomedial trajectory across the superior cerebellar peduncle and carry the highest risk, while lesions reaching the floor of the fourth ventricle or crossing the axial midline are less favorable for this corridor [16]. All lesions in the present cohort were centrally located within the pons with extension into the MCP and/or tegmentum but without contralateral midbrain involvement, corresponding to the favorable end of this spectrum and consistent with the gross-total resection and low permanent morbidity observed.

Several skull base approaches have been described to access the lateral and ventral brainstem, including the subtemporal, combined supra- and infratentorial (presigmoid transtentorial), translabyrinthine, retrolabyrinthine, retrosigmoid, and far-lateral (transcondylar) approaches [3, 4, 12, 24, 26, 29, 32]. In intrinsic brainstem lesions, the surgical corridor determines the working angle and trajectory length, whereas the entry zone determines which neural structures are traversed. The latter is therefore particularly important because it largely determines the potential neurological morbidity of the procedure.

The MCP can be reached through several corridors, including the retrosigmoid approach used in our series, an extended retrosigmoid approach, and the presigmoid retrolabyrinthine approach. The retrolabyrinthine route provides a shorter trajectory and a more favorable angle of attack, but requires temporal bone drilling and additional time for the approach [2]. We selected the retrosigmoid corridor because splitting the horizontal (petrosal) fissure provides sufficient exposure of the posterolateral surface of the middle cerebellar peduncle without the need for petrous bone removal [19, 23, 27].

The most relevant approaches for comparison with the trans-MCP route are the established safe entry zones of the pons. On the anterolateral surface, these include the peritrigeminal (supratrigeminal) zone, located rostral to the trigeminal root entry zone, and the infratrigeminal (lateral pontine) zone, located between the trigeminal root and the facial-vestibulocochlear complex. On the dorsal surface, the suprafacial and infrafacial triangles of the floor of the fourth ventricle are commonly used, while the median sulcus may be considered for selected midline lesions [10].

Each entry zone has a characteristic pattern of potential neurological deficits, reflecting the structures encountered along the surgical trajectory. The supra- and infratrigeminal zones provide a short and direct route to ventrolateral pontine lesions but it is close to the trigeminal fibres and, more importantly, the corticospinal tract. Trigeminal sensory disturbances and hemiparesis are therefore important potential complications. The infratrigeminal zone has been advocated particularly for lateral pontine lesions because it may provide access while avoiding the abducens and facial nuclei, which are more vulnerable when the floor of the fourth ventricle is traversed [4]. Dorsal entry zones provide direct access to lesions that reach the fourth ventricle but carry a recognized risk of abducens and facial nerve palsy as well as internuclear ophthalmoplegia.

The MCP differs from these pontine entry zones because it consists predominantly of transverse pontocerebellar and cerebellar afferent fibres rather than cranial nerve nuclei or major descending motor pathways. Consequently, injury to the MCP is expected to produce predominantly cerebellar symptoms, such as ataxia, dysarthria, vertigo, and gaze-evoked nystagmus, rather than primary motor or oculomotor deficits [16, 18]. The postoperative deficits observed in our five patients were consistent with this anatomical consideration, whereas the trigeminal and facial hypesthesia observed in three patients reflects the lateral pontine entry in proximity to the trigeminal root-entry zone and the lateral sensory pathways. No patient developed a new hemiparesis or gaze palsy, and the preoperative hemiparesis improved in all patients. The corridor-related deficits were well tolerated and largely transient, regressing over follow-up. This concordance between the deficit profile and the anatomy of the corridor underscores the physiological logic of the approach: morbidity reflects the price of peduncular transgression rather than injury to eloquent pontine grey matter or descending motor pathways.

The different entry zones should therefore not be regarded as interchangeable. Their selection should be based primarily on the anatomical relationship between the lesion and the brainstem surface rather than on individual surgical preference. In the classification proposed by Catapano and colleagues, pontine cavernous malformations are categorized according to their relationship to the brainstem surface, and the recommended entry zone follows from this anatomical presentation. Peritrigeminal and MCP lesions are generally approached laterally, whereas rhomboid lesions extending to the floor of the fourth ventricle are more appropriately approached dorsally [6, 7]. The same group demonstrated that deep lesions without a surface presentation carry a higher risk of new postoperative neurological deficits than superficial or exophytic lesions, regardless of the entry zone selected [5]. Our findings are consistent with these observations. All lesions in our series were deep and intrinsic, and all patients developed some new postoperative neurological deficit. Importantly, however, these deficits were predominantly related to the surgical corridor rather than to injury of the pontine core. An additional finding from our series is that deficits resulting from transgression of the MCP were generally well tolerated and showed substantial reversibility, which may contrast with the potentially more disabling deficits associated with injury to the dorsal pontine entry zones.

These observations should nevertheless be interpreted cautiously. The comparison with other entry zones is indirect and based on series with different patient populations, lesion characteristics, and surgical strategies. Given the small number of patients in our series, no definitive conclusion regarding the relative safety of the trans-MCP approach compared with other pontine entry zones can be drawn.

However, the present multicenter experience reinforces the underlying anatomical and technical rationale and supports the retrosigmoid trans–MCP approach as a viable surgical strategy for intrinsic pontine cavernous malformations. Despite the deep-seated location of all lesions and the absence of a pial or ependymal surface, gross total resection was achieved without mortality and with low overall morbidity. Postoperative neurological deficits, including cranial nerve dysfunction, were largely transient, while there was improvement in preexisting deficits. This consistent pattern of durable lesion control and favorable functional recovery underscores the feasibility of the trans-MCP approach to maximize resection while minimizing neurological injury, thereby establishing it as a reliable and reproducible technique in experienced hands.

Future refinements of the technique—such as splitting the peduncular fiber bundles along their natural orientation and integrating diffusion tensor imaging (DTI)–based tractography for trajectory planning—may further minimize fiber tract disruption and improve functional outcomes [11].

Our study has several limitations. It is a retrospective analysis of only five patients. The cohort is therefore too small for any statistical analysis and also too small to detect rare but severe complications. There was no control group, and a comparison with other safe entry zones, with other corridors to the MCP or with conservative treatment was not possible. The follow-up was relatively short and not uniform, which is not sufficient to judge late functional recovery, delayed rebleeding or the durability of the resection.

## Conclusion

The trans-MCP approach combines anatomical logic, reproducibility, and safety for centrally located pontine cavernomas.

## Data Availability

The datasets generated and/or analyzed during the current study are available upon reasonable request, subject to approval by the institutional data protection committee and in accordance with applicable data protection regulations.

## Declarations

### Artificial intelligence (AI)-assisted technology

Generative AI was used solely for language refinement. All content was critically reviewed, verified, and approved by the authors, who take full responsibility for the accuracy and integrity of the final manuscript. No AI tools were used for data analysis, interpretation, or the generation of original scientific content.
